# Analysis of the Highest Altmetrics-scored Articles in Emergency Medicine Journals

**DOI:** 10.5811/westjem.21201

**Published:** 2025-02-14

**Authors:** Başak Bayram, Murat Cetin, Önder Limon, Brit Long, Michael Gottlieb

**Affiliations:** * ˙Izmir Metropolitan Municipality Eşrefpaşa Hospital, Izmir, Türkiye; † Dr. Behçet Uz Children’s Education and Research Hospital, Department of Emergency Medicine, Konak, Izmir, Türkiye; ‡ Izmir University of Economics, Faculty of Medicine, Medicalpoint Hospital, Department of Emergency Medicine, Karşıyaka, Izmir, Türkiye; § San Antonio Uniformed Services Health Education Consortium, Department of Emergency Medicine, Fort Sam Houston, Texas; ∥ Rush University Medical Center, Department of Emergency Medicine, Chicago, Illinois

## Abstract

**Introduction:**

Alternative metrics (altmetrics) have emerged as invaluable tools for assessing the influence of scholarly articles. In this study we aimed to evaluate correlations between Altmetric Attention Scores (AAS), and sources and actual citations in articles displaying the highest AAS within emergency medicine (EM) journals.

**Methods:**

We conducted an analysis of EM journals listed in the Science Citation Index Expanded (SCIE) using the Altmetric Explorer tool. We analyzed the journals that received the highest number of mentions, the sources of AAS, the regions most frequently mentioned, and the geographical distribution of mentions. In the subsequent stage of our analysis, we conducted an examination of the 200 top-ranked articles that had received high AAS and were published in SCIE EM journals from January 1, 2013–January 1, 2023. We sought to determine the correlations between the AAS and the citation counts of articles on Google Scholar and the Web of Science (WOS).

**Results:**

Of 40,840 research outputs evaluated, there were 510,047 shares across multiple platforms. The AAS were present for 36,719 articles (89.9%), while 10.1% had no score. In the review of the top 200 articles with the highest AAS, the median score was 382.5 (interquartile range 301.3–510.8). Of the research output evaluated, 38% were observational studies, 13% case reports, and 13% reviews/meta-analyses. The most common research topics were emergency department (ED) management and COVID-19. There was no correlation between AAS and WOS citation numbers (r_s_ = −0.041, *P* = 0.563, 95% confidence interval [CI] −0.175–0.087). There was a weak correlation identified between WOS citations and mentions on X, and a moderate correlation observed for WOS citations and blog mentions (r_s_ = 0.330, *P* < .001, 95% CI 0.174 to 0.458; r_s_^2^ = 0.109, and r_s_ = 0.452, *P* < .001, 95% CI 0.320–0.566; and r_s_^2^ = 0.204, respectively). However, we found a strong positive correlation between WOS citations and the number of Mendeley readers (r_s_ = 0.873, *P* < .001, 95% CI 0.82–0.911, r_s_^2^ = 0.762).

**Conclusion:**

While most articles in EM journals received an AAS, we found no correlation with traditional citation metrics. However, Mendeley readership numbers showed a strong positive correlation with citation counts, suggesting that academic platform engagement may better predict scholarly impact.

Population Health Research Capsule What do we already know about this issue?

*Altmetrics emerged in the 2010s to address the limitations of traditional citation-based metrics in evaluating research impact.*

What was the research question?

*We explored the correlations between Altmetric Attention Scores, their sources, and citation counts for articles published in EM journals.*

What was the major finding of the study?

*No correlation was found between AAS and Web of Science; it was weak for X mentions, moderate for blogs, but strong for Mendeley readership (r_s_ = 0.873, 95% CI 0.822–0.911).*

How does this improve population health?

*Focusing on article dissemination through individual altmetric sources, rather than total scores, can help researchers more effectively reach their target audiences.*

## INTRODUCTION

Alternative metrics (altmetrics) emerged in the early 2010s in response to the limitations of traditional citation-based metrics.^1^ Altmetrics use a broader set of indicators such as page views, downloads, social media mentions, news media coverage, and expert recommendations to provide a more comprehensive understanding of an article’s influence.^2^ Platforms like Altmetric.com and Plum Analytics provide tools for evaluating the reach and impact of scholarly articles, helping to track their online dissemination in real time. An increasing amount of evidence indicates that maintaining an active online presence can directly influence a researcher’s credentials as evaluated by conventional measures.^3^ By considering various aspects beyond citations alone, altmetrics provides researchers and institutions with a more holistic assessment of their work’s societal impact. While the purpose of these metrics is to measure social impact, early social media visibility after publication can also increase and predict citations.^4^ Assessing these activities could provide faster evaluations of an article’s impact and predict citations, serving as an early identifier for emerging areas of research growth.^5^

Within the medical field specifically, these metrics offer insights into both scholarly recognition and public reception of research findings. Moreover, the relationship between these metrics underscores the changing landscape of scholarly communication, as researchers, clinicians, and the public alike engage with and contribute to the dissemination of research findings through online platforms. With the growth of digital communication and social media, the speed and the scale of information-sharing have accelerated, making altmetrics an invaluable tool for assessing real-time impact of articles.^6^ Emerging trends can be key to a more efficiently functioning field of medicine.^7^

Emergency medicine (EM) thrives on the timely dissemination of research and information that directly impacts patient care.^8^ The first altmetrics analysis in EM conducted by Barbic et al found that the most-cited articles on social media in EM from 2011 were often published in non-EM biomedical journals.^9^ Although this may suggest that authors in the field of EM select high-impact journals to increase the effectiveness of their publications, altmetric scores and journal impact factors are not correlated.^10^ The social impact of an article may be better assessed by focusing on individual altmetric score sources rather than the overall score.^11^ This was supported by a recent study that found a direct correlation between X (formerly Twitter) mentions and article citations among EM research.^12^ Our aim in this study was to investigate the correlations between the altmetric scores, their sources, and citations.

## METHODS

In the first stage of the study, we used the Altmetric Explorer tool from Altmetric.com (Altmetric LLP, London, UK) to assess the Altmetric Attention Scores (AAS) of scholarly articles published in EM journals indexed in the Science Citation Index Expanded (SCIE). The AAS is a metric that evaluates the attention a research output receives using a weighted system that assigns distinct values to various sources, such as news outlets, blogs, and social media platforms. Sources with greater impact, such as news articles, are attributed higher weights compared to social media mentions. The score is calculated by a sophisticated algorithm that factors in not only the number of mentions but also variables such as duplicate posts and the credibility of the news sources.^13^

All Altmetric Explorer assessments were conducted and downloaded as a CSV file on September 2, 2023. The initial analysis focused on examining the distribution of altmetric data by country and journal, as well as evaluating the sources of AAS scores over time. The 2022 Journal Citation Indicator (JCI) scores for the journals were obtained from the Web of Science (WOS) Master Journal List. These JCI scores represent the average citation impact of articles published between 2019–2021.

In the second stage we identified the top 200 articles with the highest AAS published in SCIE EM journals between January 1, 2013–January 1, 2023. We assessed the AAS of these articles along with the sources of their mentions. Citation counts for these articles were evaluated using both WOS and Google Scholar. To calculate the annual citation number, we divided the total citation counts obtained from the search engines by the number of years since the articles’ publication. We obtained full-text access for the articles to determine the article’s subject (eg, emergency department management, trauma, toxicology, resuscitation, critical care, COVID-19) and type (randomized controlled trial, observational study, case reports, reviews (systematic review and meta-analysis). Additionally, we determined the country of the first-named author of each article.

We assessed the correlation between the AAS and citation counts of the articles. Furthermore, two EM specialists from the research team independently reviewed the AAS and all screening processes of the study. In cases where the two evaluators did not reach a consensus, a third EM specialist conducted the evaluation. The study was approved by the Local Ethics Committee of İzmir Provincial Health Directorate Dr. Behcet Uz Pediatric Diseases and Surgery Training and Research Hospital.

### Statistical Analysis

We performed statistical analysis using SPSS 29.0 for Windows (SPSS Statistics, IBM Corp, Armonk, NY). Categorical variables were evaluated using the Kolmogorov-Smirnov test. Among the variables, those that fit the normal distribution were presented as the mean ± standard deviation, and those that did not fit the normal distribution were presented as median (interval) or median (interquartile range [IQR]). We used Mann-Whitney U and Kruskal-Wallis tests to compare numerical variables. Spearman correlation analysis was used to assess the relationship between AAS, their sources (mentions), and WOS and Google Scholar citation numbers. Spearman correlation analysis was used to analyze distributions of AAS, the source of the scores (mentions), and WOS and Google Scholar citation numbers. We interpreted the correlations as weak, moderate, strong, and very strong based on the resulting coefficients.^14^ Statistical significance was recognized when P < 0.05.

## RESULTS

Of the 40,840 research outputs evaluated, 510,047 were shared across multiple platforms. Altmetric Attention Scores were present for 36,719 articles (89.9%), while 10.1% had no score. The online engagement for this content included 459,391 tweets from 114,708 unique tweeters in 206 countries, 14,355 Facebook posts on 2,141 unique pages in 60 countries, 19,571 news stories by 1,988 unique outlets in 86 countries, and 2,791 policy documents from 79 unique sources in 18 countries. The top 10 journals had no mentions on Pinterest, Syllabi, or LinkedIn. Only two journals received mentions on the Chinese microblogging platform Weibo, each mentioned once. Figure illustrates the frequency of mentions corresponding to the publication years.

**Figure.**
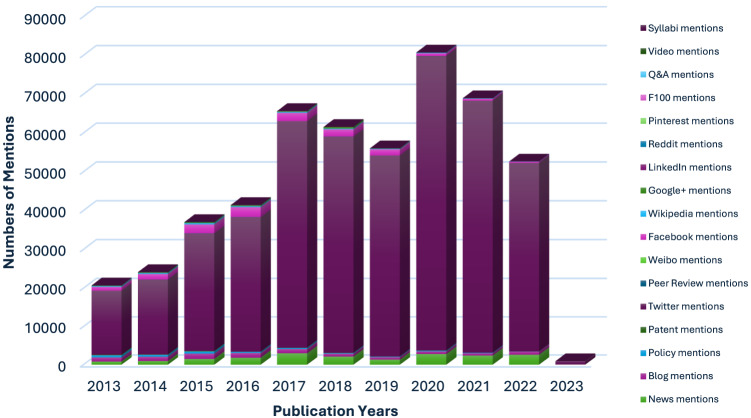
The number of mentions in altmetric score resources over publishing years.

The Altmetrics–X demographics data revealed the top five countries contributing to mentions for the analyzed content. The country was not specified in 193,148 posts (42.0%) and 54,517 profiles (47.5%). The largest number of posts were from the United States (Table 1).

**Table 1.** Country distribution of altmetrics-X demographics.

Country name	Number of posts^*^ (n [%])	Number of profiles^**^ (n [%])
Country not specified	193,148 (42.0%)	54,517 (47.5%)
Unites States	89,876 (19.6%)	18,734 (16.3%)
United Kingdom	50,504 (11.0%)	9,208 (8.0%)
Canada	27,916 (6.1%)	4,297 (3.7%)
Australia	15,307 (3.3%)	2,435 (2.1%)
Spain	13,538 (2.9%)	3,863 (3.4%)

* A post refers to an individual mention or engagement on a social media platform (such as an X mention, Facebook post, blog entry, etc) where a specific research output is shared or discussed.

** A profile refers to the unique social media account or user (eg, an X or Facebook account) that made the post or mention.

Overall, 90% of the 36,780 articles had an AAS ≥ 1. The median (IQR) values for these articles were as follows: AAS 3 (1–9), X mentions 3 (1–9); Mendeley readership 24 (9–50); Dimensions citations 5 (1–15); and blog mentions 0 (0–24). We found a strong correlation between AAS and X mentions (r_s_ = 0.712, 95% CI 0.707–0.717, *P* < .001) and a weak correlation between AAS and the number of Mendeley readers (r_s_ = 0.338 to 356, *P* < .001).

We found two journals that lacked AAS. The median total mentions for EM journals were 14,154 (ranging from 57–77,460). Mentions on X had a median of 12,284 (ranging from 56–68,078), Facebook mentions had a median of 234 (ranging from 0–2,180), blog mentions had a median of 126.5 (ranging from 0–1,866), and news mentions had a median of 288 (ranging from 0–5,040).

The EM journals with the highest total number of mentions were *Annals of Emergency Medicine, Resuscitation, and the American Journal of Emergency Medicine*. Conversely, the AAS per article was highest for *Academic Emergency Medicine, Annals of Emergency Medicine, and the Emergency Medicine Journal*, respectively (Table 2). We found a moderate correlation (r_s_ = 0.518, *P* = 0.07, 95% CI 0.152–759) between the 2022 JCI and the total number of mentions.

**Table 2.** Journals with the highest total number of mentions.

Journal title^δ^	JCI^Δ^	Total number of mentions	Total number of mentions per article	News	Blog	Policy	Patent	X	Peer review	Facebook	Wikipedia	Google+	Reddit	F1000	Q&A	Video
Academic Emergency Medicine	1.56	46,945	23.97	2,332	1,109	199	58	41,615	10	1,178	99	258	22	5	0	61
Annals of Emergency Medicine	2.3	77,460	20.85	5,040	1,866	221	58	68,078	19	1,830	136	107	40	16	0	51
Emergency Medicine Journal	1.2	46,196	16.92	1,025	1,094	159	16	42,303	2	1,374	80	114	18	2	0	10
Resuscitation	1.87	64,941	15.08	1,532	845	400	151	59,341	1	2,180	149	218	32	29	3	61
The Western Journal of Emergency Medicine	1.06	20,345	14.91	793	390	106	28	18,298	3	520	101	81	2	3	0	21
Canadian Journal of Emergency Medicine	0.75	25,414	14.88	265	390	177	15	23,791	1	620	107	37	5	1	0	7
Scandinavian Journal of Trauma, Resuscitation and Emergency Medicine	1.32	16,901	14.81	224	145	48	18	16,021	3	286	49	75	19	3	0	10
Journal of Emergency Medicine	1.2	35,634	12.66	1,298	798	147	33	32,356	3	631	188	94	28	5	2	51
American Journal of Emergency Medicine	1.47	50,028	9.63	2,156	1,190	335	55	44,442	16	1,502	172	68	17	10	1	64
Pediatric Emergency Care	0.77	16,921	5.52	308	430	97	6	14,434	4	1,534	43	12	0	4	0	50

Δ JCI: Journal Citation Indicator.

δ The journals are ranked based on the total mentions per article.

In the review of the top 200 articles with the highest AAS, the median score of the articles was 382.5 (IQR 301.3–510.8). The AAS with sources and number of citations of the top 50 articles are provided in Table 3. The median WOS citations for the articles was 16 (IQR 5–39), with an annual citation count of 4.7 (IQR 1.8–8.9). The median Google Scholar citations was 29 (IQR 11–65), with an annual citation count of 7.5 (IQR 3.2–15.1). Among these articles, 38% were observational studies, while case reports and reviews/meta-analyses constituted 13%. The most common research topics were ED management and COVID-19 (Table 4).

**Table 3.** Top 50 articles with highest altmetric scores.

Number	Title	Journal	Year	News mentions	Blog mentions	X mentions	Facebook mentions	Wikipedia mentions	Number of mendeley readers	Number of dimensions citations	Altmetric attention score	WOS citations	Google Scholar citations
1	N95 respirator cleaning and reuse methods proposed by the inventor of the N95 mask material	*J Emerg Med*	2020	120	2	4848	1	0	162	48	2621	39	67
2	Association between delays to patient admission from the emergency department and all-cause 30-day mortality	*Emerg Med J*	2022	156	6	2467	0	1	100	39	2040	25	59
3	Excited delirium: a systematic review	*Acad Emerg Med*	2017	447	3	190	8	2	120	49	1516	39	65
4	Vaccine-induced myocarditis in two intern doctors in the same night shift	*Prehosp Disaster Med*	2022	0	0	2868	1	0	7	3	1430	0	3
5	Loperamide abuse associated with cardiac dysrhythmia and death	*Ann Emerg Med*	2017	161	8	203	13	2	96	80	1319	66	103
6	A lay perspective and commentary on the association between delays to patient admission from the emergency department and all-cause 30-day mortality	*Emerg Med J*	2022	162	2	47	1	0	2	1	1269	1	1
7	Characteristics of paediatric out-of-hospital cardiac arrest in the United States	*Resuscitation*	2020	152	0	50	0	0	33	20	1173	17	23
8	A coronavirus disease 2019 (COVID-19) patient with bilateral orchitis	*Am J Emerg Med*	2021	23	2	1707	1	0	72	38	1128	23	45
9	The association of treatment with hydroxychloroquine and hospital mortality in COVID-19 patients	*Intern Emerg Med*	2020	1	1	3650	0	0	130	24	1096	18	29
10	Priapism in a patient with coronavirus disease 2019 (COVID-19)	*Am J Emerg Med*	2021	101	3	530	0	0	100	35	1083	22	50
11	Esophageal rupture after ghost pepper ingestion	*J Emerg Med*	2016	124	8	143	11	4	22	4	1021	3	5
12	Accidental occupational exposure to a large volume of liquid fentanyl on a compromised skin barrier with no resultant effect	*Prehosp Disaster Med*	2022	27	2	1228	2	0	8	1	999	1	3
13	Vitamin D deficiency is associated with higher risks for SARS-CoV-2 infection and COVID-19 severity: a retrospective case–control study	*Intern Emerg Med*	2022	5	1	1469	0	0	48	19	994	13	20
14	Characterization of in-flight medical events involving children on commercial airline flights	*Ann Emerg Med*	2020	132	1	31	4	0	57	10	963	8	13
15	Interrogation of patient smartphone activity tracker to assist arrhythmia management	*Ann Emerg Med*	2016	110	9	169	10	0	103	33	963	20	46
16	AWARE—AWAreness during REsuscitation—A prospective study	*Resuscitation*	2014	113	19	269	123	8	357	133	946	93	258
17	Alarming trends in US domestic violence during the COVID-19 pandemic	*Ann Emerg Med*	2020	127	10	53	0	2	542	507	929	356	732
18	Use of antibiotic coated intramedullary nails in open tibia fractures: a European medical resource use and cost-effectiveness analysis	*Injury*	2021	127	0	5	0	0	19	12	924	7	12
19	Bilateral retinal detachments in a healthy 22-year-old woman after Moderna SARS-COV-2 vaccination	*J Emerg Med*	2021	0	0	3901	0	0	45	12	922	12	16
20	Stopping haemorrhage by application of rope tourniquet or inguinal compression (SHARC study)	*Emergency Medicine Australasia*	2021	198	2	37	0	0	6	0	915	0	0
21	The use of the word “quiet” in the emergency department is not associated with patient volume: a randomized controlled trial	*Am J Emerg Med*	2022	1	3	1521	3	0	25	2	879	2	3
22	Aromatherapy versus oral ondansetron for antiemetic therapy among adult Emergency department patients: a randomized controlled trial	*Ann Emerg Med*	2018	5	10	1968	13	0	139	19	832	3	29
23	Cyclic vomiting presentations following marijuana liberalization in Colorado	*Acad Emerg Med*	2015	103	8	60	9	1	104	95	825	72	120
24	Removal of iliosacral screws: the washer problem	*Injury*	2021	112	0	10	0	0	2	0	819	0	0
25	Persistent hiccups as an atypical presenting complaint of COVID-19	*Am J Emerg Med*	2020	57	1	2774	0	0	146	27	813	26	54
26	It isn’t like this on TV: revisiting CPR survival rates depicted on popular TV shows	*Resuscitation*	2015	91	10	117	0	0	131	53	808	45	78
27	In-hospital cardiac arrest outcomes among patients with COVID-19 pneumonia in Wuhan, China	*Resuscitation*	2020	7	6	1369	4	0	440	239	802	178	332
28	Identifying safe corridors for anterior pelvic percutaneous instrumentation using computed tomography-based anatomical relationships	*Injury*	2022	167	0	0	0	0	2	0	792	0	0
29	Risk of acute kidney injury after intravenous contrast media administration	*Ann Emerg Med*	2017	54	24	540	14	0	414	187	789	167	260
30	Acute kidney injury after computed tomography: a meta-analysis	*Ann Emerg Med*	2018	49	12	638	5	3	303	127	785	100	175
31	Trends in inequities in the treatment of and outcomes for women and minorities with myocardial infarction	*Ann Emerg Med*	2022	133	2	12	0	0	8	5	763	2	6
32	Single versus dual incision approaches for dual plating of bicondylar tibial plateau fractures have comparable rates of deep infection and revision surgery	*Injury*	2022	180	0	0	0	0	1	2	737	0	2
33	Comparison of oral ibuprofen at three single-dose regimens for treating acute pain in the emergency department: a randomized controlled trial	*Ann Emerg Med*	2019	6	3	1160	6	0	104	30	713	28	41
34	Young woman with paraplegia following a motor vehicle crash	*Ann Emerg Med*	2016	0	0	1042	12	0	5	0	698	0	0
35	Association between the opening of retail clinics and low-acuity emergency department visits	*Am J Emerg Med*	2017	96	7	52	0	0	46	31	660	25	50
36	STEMI mimic: focal myocarditis in an adolescent patient after mRNA COVID-19 vaccine	*J Emerg Med*	2021	1	3	2112	0	0	58	13	644	10	15
37	One-year mortality of patients after emergency department treatment for nonfatal opioid overdose	*Ann Emerg Med*	2020	36	14	563	2	0	134	126	644	103	157
38	Academic emergency medicine physicians’ anxiety levels, stressors, and potential stress mitigation measures during the acceleration phase of the COVID-19 pandemic	*Acad Emerg Med*	2020	79	4	16	0	0	286	117	605	86	168
39	Bystander CPR is associated with improved neurologically favourable survival in cardiac arrest following drowning	*Resuscitation*	2017	81	0	28	3	0	64	36	603	35	49
40	Cold anaphylaxis: a case report	*J Emerg Med*	2020	86	11	11	0	0	9	0	594	0	0
41	Hospital volume and post-arrest care: a complex topic with more questions than answers	*Resuscitation*	2017	75	0	1	0	0	8	0	578	0	0
42	The impact of race and disease on sickle cell patient wait times in the emergency department	*Am J Emerg Med*	2013	64	5	36	1	0	97	93	572	77	153
43	Gender disparities in the application of public-access AED pads among OHCA patients in public locations	*Resuscitation*	2020	0	0	4546	0	0	36	7	564	5	9
44	Are there disparities in the location of automated external defibrillators in England?	*Resuscitation*	2022	70	1	14	1	0	28	8	564	6	13
45	United States 2020 emergency medicine resident workforce analysis	*Ann Emerg Med*	2022	73	1	20	0	0	14	4	563	3	5
46	Expert consensus guidelines for stocking of antidotes in hospitals that provide emergency care	*Ann Emerg Med*	2018	53	0	231	8	0	132	46	560	40	69
47	Longitudinal trends in U.S. drug shortages for medications used in emergency departments (2001–2014)	*Acad Emerg Med*	2015	78	9	38	1	0	39	35	554	26	48
48	Avoiding potential harm by improving appropriateness of urinary catheter use in 18 emergency departments	*Ann Emerg Med*	2014	68	0	4	0	0	58	19	534	16	30
49	Rapid adoption of low-threshold buprenorphine treatment at California emergency departments participating in the CA Bridge Program	*Ann Emerg Med*	2021	63	3	60	0	0	75	37	532	31	40
50	Cool running water first aid decreases skin grafting requirements in pediatric burns: a cohort study of two thousand four hundred ninety-five children	*Ann Emerg Med*	2020	28	4	444	7	0	76	37	511	25	51

**Table 4.** The citations are categorized based on the topics and types of the top 200 articles with the highest Altmetric Attention Scores.

	n (%)	Altmetric attention scores (median/IQR)	Number of WOS citations (median/IQR)	Number of scholar citations (median/IQR)
Article type				
Observational studies	76 (38)	432.5 (337.8–560.8)	18.0 (7.3–45.5)	30.0 (12.3–63.5)
Reviews	26 (13)	323.0 (272.5–386.8)	30.5 (7.0–72.3)	53.0 (11.8–130.8)
Case reports	26 (13)	433.0 (295.8–972.0)	11.5 (1.8–24.5)	21.0 (3.8–47.0)
Randomized controlled trials	21 (10.5)	330.0 (279.5–408.0)	21.0 (7.5–33.0)	40.0 (25.5–69.5)
Guidelines	7 (3.5)	280.0 (257.0–340.0)	35.0 (14.0–40.0)	69.0 (28.0–79.0)
Other	44 (22)	387.0 (344.0–475.5)	9.0 (4.0–29.5)	15.5 (4.3–48.3)
Topics				
ED management	39 (19.5)	369.0 (295.0–452.0)	21.0 (9.0–41.0)	36.0 (16.0–66.0)
COVID-19	29 (14.5)	427.0 (327.5–925.5)	22.0 (10.5–91.5)	36.0 (15.5–163.0)
Toxicology	24 (12.0)	440.5 (340.8–623.0)	27.5 (4.0–44.8)	42.0 (8.8–71.3)
Trauma	23 (11.5)	319.0 (261.0–468.0)	5.0 (0.0–10.0)	7.0 (2.0–24.0)
Critical care	19 (9.5)	425.0 (347.0–578.0)	19.0 (7.0–56.0)	34.0 (15.0–78.0)
Other	66 (33.0)	378.0 (299.0–489.8)	5.0 (0.0–16.0)	29.0 (10.0–62.8)
Analysis of COVID-19 articles				
COVID-19	29 (14.5)	427.0 (327.5–925.5)	22.0 (10.5–91.5)	36.0 (15.5–163.0)
Non-COVID-19	171 (75.5)	380.0 (298.0–485.0)	16.0 (5.0–37.0)	29.0 (9.0–64.0)

*ED*, emegency department; *IQR*, interquartile range; *WOS*, Web of Science.

The AAS of reviews and guidelines were lower, while observational studies and case presentations were higher (*P* = 0.02). The AAS was higher for topics related to toxicology, COVID-19, and critical care (*P* = 0.02). While there was an increase in AAS and WOS citations in COVID-19-related papers, these variations were not significant (*P* = 0.09 and *P* = 0.08, respectively).

There was no significant correlation between AAS and WOS citation numbers (r_s_ = −0.041, *P* = 0.56, 95% CI −0.175–0.087, r_s_^2^ = 0.0017) or Google Scholar citations (r_s_ = −0.038, *P* = 0.59, 95 % CI −0.174–0.101, r_s_^2^ = 0.0014). However, there was a very strong positive correlation between WOS and Google Scholar citation numbers (r_s_ = 0.973, *P* < .001, 95% CI 0.955–0.984, r_s_^2^ = 0.947). Despite the weak correlation identified between WOS citations and X mentions, and the moderate correlation observed for blog mentions (r_s_ = 0.330, *P* < .001, 95 % CI 0.174 to 0.458, r_s_^2^ = 0.109, and r_s_ = 0.452, *P* < .001, 95% CI 0.320 to 0.566, r_s_^2^ = 0.204, respectively), there was a very strong positive correlation observed in the number of Mendeley readers. (r_s_ = 0.873, *P* < .001, 95% CI 0.822–0.911, r_s_^2^ = 0.762). No correlation was observed between news mentions (r_s_ = −0.107, *P* = 0.10, 95% CI −0.246–0.046, r_s_^2^ = 0.0012), and video mentions (r_s_ = 0.037, *P* = 0.60, 95% CI −0.078 to 0.145, r_s_^2^ = 0.0013).

## DISCUSSION

This study examined altmetrics of EM journal articles from the 2013–2023. Ten percent of EM journal articles were never mentioned on social media. Compared to Barbic et al’s investigations from 2011, AAS for the most cited publications have increased significantly in the subsequent decade.^9^ Social media followers for EM journal articles have increased significantly in recent years. Interestingly, three-quarters of the 200 most-cited articles were published after this study, with 45% published after the COVID-19 pandemic. Social media followers for EM journal articles have increased significantly in recent years.

Kolahi et al identified a weak but positive correlation between AAS and citations in their meta-analysis; the authors emphasized the importance of continuing to examine the temporal dynamics of this relationship.^15^ In our study, although no correlation was found between AAS and traditional citation counts, we observed a weak correlation between AAS and X mentions, and a moderate correlation between AAS and blog mentions. Notably, there was a very strong correlation observed between AAS and the Mendeley readership numbers.

The AAS is calculated based on the source and frequency of sharing. In this calculation, news, blog mentions, Wiki pages, policy documents, and patents have the most weight, while X has less weight. Mendeley readership and citations are not considered.^16^ It should be noted that the primary purpose of altmetrics is to measure social interest in a given topic, rather than to predict the potential citation count of an article. Traditional citations remain the gold standard for academic recognition. However, the relationship between social media and citations supports the positive impact of researchers and scientific journals using social media to enhance the visibility and influence of their articles. Incorporating the impact of social media into the gold standard of citation counts could be a way to acknowledge this evolving landscape.

The results of a study examining the impact of promoting Cochrane systematic reviews in the field of pediatric EM using X and blog posts revealed a significant increase of 10 times in the AAS of the reviews.^17^ The distribution pattern of articles on social media might vary based on the nature of the sharing. Infographics are visual representations of data meant to enhance engagement and streamline the key elements of a given study.^18^ Some data suggests that presenting research findings visually on social media may lead to a 5–7 times higher number of interactions compared to studies without visual content.^19^ ^,^ ^20^ Although using visual presentations to share results can reach a larger audience, this effect may only apply to specific areas of expertise.

The extent to which sharing influences the number of downloads and citations of a paper remains uncertain.^21^ However, altmetrics today play a crucial role as markers for assessing the spread of content via social media to reach the intended audience. Temporal patterns in article altmetrics exhibit variation across different data sources. A study investigating altmetrics temporal trends reported that X engagement started and ended quickly, while Mendeley readership increased steadily over the next few years.^22^ An excellent way to maintain interest in published articles is to use altmetrics data sources in combination with methods that engage the target audience and regularly update the content.

During the COVID-19 pandemic, there was a notable increase in the dissemination of information on social media platforms, with healthcare professionals using these platforms more frequently. Our analysis shows that nearly half of the top 200 publications in EM journals were published after the onset of the pandemic. Additionally, when evaluating the comprehensive altmetrics of articles in EM journals, we found there was a clear rise in mentions during 2020–2021. The evaluation undertaken in this study encompasses references made until the start of 2023. While future studies will determine whether this upward trend will persist, it is foreseeable that the surge in researchers using social media to monitor scientific information will continue as a result of the COVID-19 pandemic. This rise can be linked to the surge in sharing activities associated with COVID-19. Nevertheless, our investigation found no discernible distinction between AAS and WOS citations when comparing papers linked to COVID-19 and those unrelated to it.

## LIMITATIONS

This study has several limitations, particularly its emphasis on quantitative data analysis and the use of a single data source. The current Altmetric database mainly emphasizes the number of mentions, and our analysis exclusively compared these metrics. In approximately 50% of the mentions on X we were unable to determine the country associated with the account. In addition to the increased interaction of the attributes of social media shares, it is more important to evaluate the relationship of the information to the target audience, reference, and download.^20^ In the early part of the study, we analyzed all articles published in EM journals, but we made comparisons with traditional references for only 200 articles. In contrast to previous studies, we evaluated articles with the highest AAS instead of the altmetrics of the most cited articles in traditional reference indices. Another limitation of this study is the inability to fully differentiate the impact of increased social media usage during the COVID-19 pandemic on AAS. The surge in online content and interactions during the pandemic may have artificially inflated AAS values, particularly for articles published during this period, potentially affecting the relationship between AAS and traditional citation counts.

## CONCLUSION

There has been a notable rise in Altmetrics Attention Scores in recent years, driven by increased use of social media for following scientific research, particularly during the COVID-19 pandemic. Articles focusing on toxicology, COVID-19, and resuscitation/critical care tend to receive the highest AAS. While no correlation was found between total AAS and citation counts from WOS and Google Scholar, there is a strong positive correlation between WOS citations and the number of Mendeley readers. Additionally, weak and moderate correlations were observed for mentions on X and blogs, respectively. Further research is needed to explore the relationship between altmetrics and traditional citation metrics, as well as the impact of social media on academic research visibility in EM.
